# Maximizing Aesthetic Outcomes in Delayed Breast Reconstruction: the Be.A.U.T-I.F.U.L. DIEP^®^ Step-by-Step Inset Technique

**DOI:** 10.1007/s00266-024-04502-3

**Published:** 2024-12-02

**Authors:** Marco Morelli Coppola, Clara Schaffer, Giulio Jad Jaber, Gianluca Sapino, Pietro Giovanni di Summa

**Affiliations:** 1 Operative Research Unit of Plastic, Reconstructive and Aesthetic Surgery, Fondazione Policlinico Universitario Campus Bio-Medico, Rome, Italy; 2 Department of Plastic and Hand Surgery, Centre Hospitalier Universitaire Vaudois (CHUV), University of Lausanne (UNIL), Rue du Bugnon 46, 1011 Lausanne, Switzerland; 3 Unit of Plastic, Reconstructive and Aesthetic Surgery, Department of Human Pathology, Policlinico Gaetano Martino, University of Messina, Messina, Italy

**Keywords:** Free tissue flaps, Microsurgery, Mastectomy, Mammaplasty, Breast neoplasms, Esthetics, Patient satisfaction

## Abstract

**Introduction:**

In microvascular breast reconstruction, the focus has shifted to achieving aesthetically pleasing results. Delayed breast reconstruction poses challenges such as ensuring natural ptosis and avoiding a “patch” effect. The Be.A.U.T-I.F.U.L. deep inferior epigastric perforator (DIEP) flap inset, presented here, offers a systematic and sequential seven-step method to optimize breast reconstruction outcomes.

**Methods:**

This approach emphasizes safety during flap harvest using the best (Be.) perforator identified via computed tomography angiography to minimize dissection and ensure solid perfusion. The flap is placed obliquely, directing its tail to the axilla (A.), enhancing upper (U.) pole volume and ptosis. The flap base is tucked-in (T-I.) to increase the projection of the breast mound, and its lateral portion is split as a fishtail (F.): the upper (U.) fin is used to define the lateral inframammary fold and prevent lateral displacement of the reconstructed breast, while the lower (L.) fin is turned under the flap to further improve projection.

**Conclusion:**

This structured approach, focusing on key breast aesthetics, ensures optimal cosmetic outcomes and can serve for most delayed microsurgical breast reconstruction scenarios.

**Level of Evidence IV:**

This journal requires that authors assign a level of evidence to each article. For a full description of these Evidence-Based Medicine ratings, please refer to the Table of Contents or the online Instructions to Authors www.springer.com/00266.

**Supplementary Information:**

The online version contains supplementary material available at 10.1007/s00266-024-04502-3.

## Introduction

**Fig. 1:**
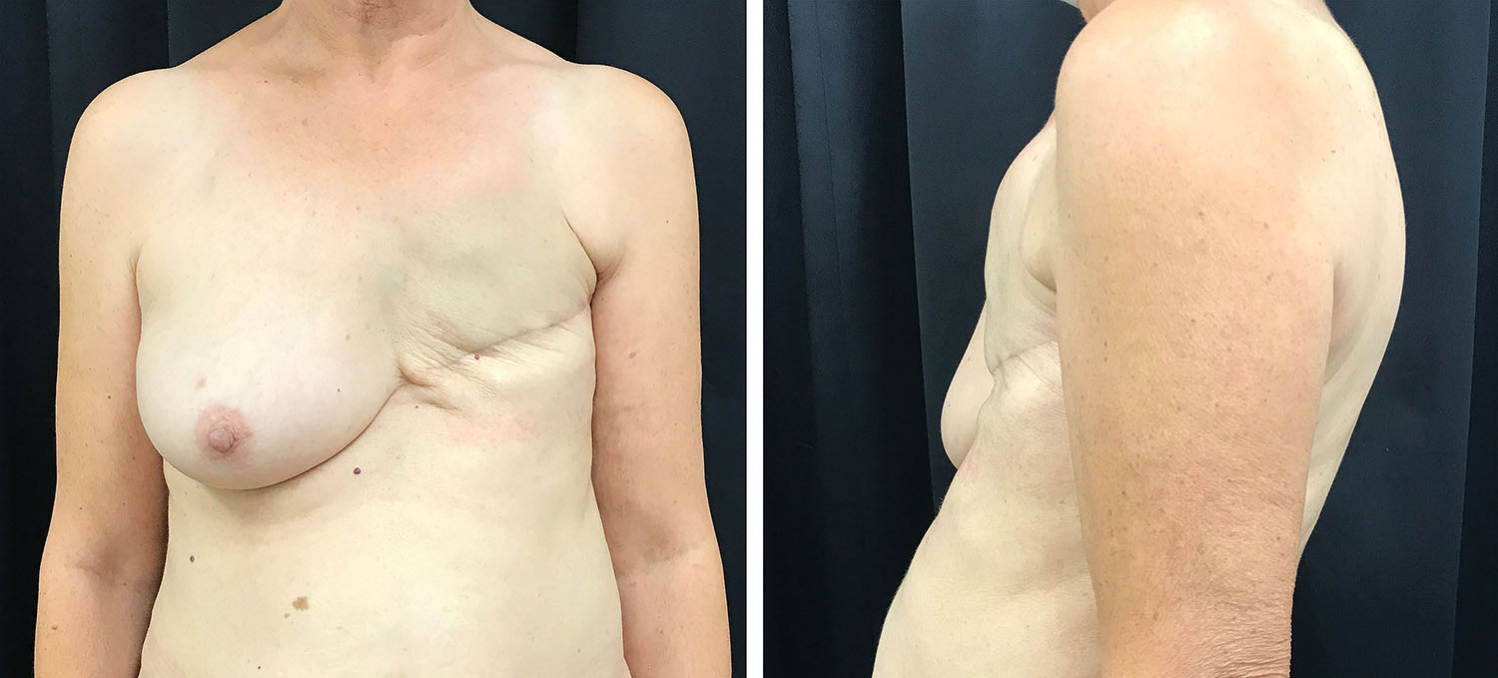
A 45-year-old patient with previous left modified radical mastectomy and adjuvant radiation therapy

In microvascular breast reconstruction, surgeons’ concerns have shifted from simple flap survival to achieving aesthetically pleasing results in order to achieve greater patients’ satisfaction [1]. In delayed breast reconstruction, for patients not undergoing immediate reconstruction or patients with previous failure of implant-based breast reconstruction, the absence of the native skin envelope sometimes makes it difficult to ensure natural ptosis and avoid a “patch” effect [2] (Fig. 1).

Blondeel described a systematic approach, highlighting the three main anatomical features of a breast to be considered when performing breast reconstruction: the footprint, the conus, and the skin envelope [3–5]. However, despite numerous publications offering solutions, there is no universal consensus on insetting techniques, which are often left to the surgeon’s taste and preferences [6–8].

The authors hereby present the Be.A.U.T-I.F.U.L. approach to DIEP flap inset, illustrating seven steps to follow in order to achieve an optimal breast footprint and match with the contralateral breast.

## Patients and Methods

The Be.A.U.T-I.F.U.L. approach prioritizes safety during flap harvest, using the single **be**st (“Be.”) perforator identified via computed tomography angiography (CTA), to minimize intramuscular dissection and ensure solid perfusion.

After revascularization, the flap is placed obliquely, directing its tail (Holm zone II) toward the **a**xilla (“A.”) with a 40–50° rotation for ipsilateral and 130–140° for contralateral perforators [9]. This inset allows for more volume in the **u**pper pole (“U.”) and ptosis than the standard horizontal inset.

Next, the base of the flap is **t**ucked-**i**n (“T-I.”), to increase projection at the midpoint of the mammary cone, where reconstructed breasts are generally deficient and may require fat grafting.

**Fig. 2:**
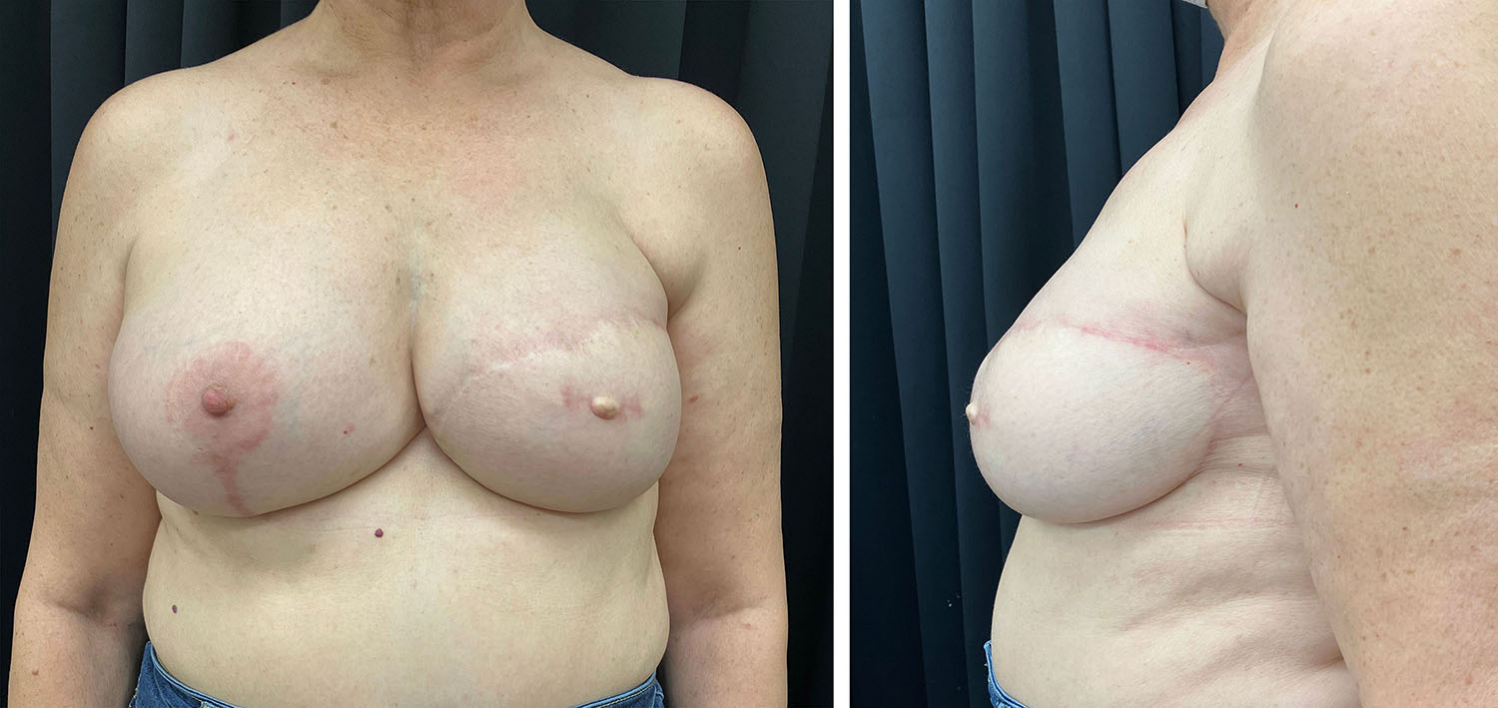
The same patient one-year post-left breast reconstruction with DIEP flap with Be.A.U.T-I.F.U.L. inset technique and contralateral mastopexy

Once the flap orientation, volume distribution, and base have been defined, the lateral portion of the flap is split like a **f**ishtail (“F.”), and both fins are de-epithelialized. The **u**pper fin (“U.”) is secured to the lateral border of the pectoralis major to define the lateral inframammary fold (IMF) and prevent lateral displacement of the flap. The **l**ower fin (“L.”) is turned under the flap and secured to the pectoralis fascia to further increase the projection of the mammary cone. (Fig. 2, Supplemental Digital Content Video)

## Discussion

Accurate preoperative planning allows effective and efficient flap harvest without hesitation amongst multiple perforators, to later focus on the inset as a critical step. Lateral row perforators offer easier and faster dissection, mainly due to the shorter intramuscular course, but are associated with a higher rate of abdominal bulge [10, 11]. Medial row perforators provide better perfusion of Holm zone III across the midline, allowing sufficiently large flaps to be raised with a relatively low risk of fat necrosis [12–14]. The selection of large-caliber perforator, regardless of the side to be reconstructed, and the use of indocyanine green angiography (ICGA) can further reduce this risk [15, 16].

Several factors should be considered to obtain a proper flap inset: body habitus, donor site, reconstruction timing, mastectomy type, and contralateral breast characteristics (width, ptosis, projection, and upper pole fullness) [17]. Although several insetting techniques have been described, a stepwise structured approach is still missing. Previous literature suggested raising the contralateral flap and to rotate it 180°, to take advantage of increased thickness of the abdominal pannus in order to provide additional volume to the lower pole. Other papers suggest a vertical inset to increase ptosis [18].

Oblique orientation offers greater freedom, compared with purely horizontal or vertical insets, providing sufficient skin and ptosis in the vertical plane while still providing sufficient width to the reconstructed breast. The key element of flap placement is the location of the flap tail (Holm zone II) in the axilla, regardless of the side of flap raise. This placement allows optimal allocation of the most consistent part of the flap in the medial quadrants and ideal ptosis adjustment by choosing the amount of flap segment between the IMF and the mastectomy scar.

Even with adequate ptosis, the reconstructed breast may lack projection and volume, in contrast to the aesthetic goal of a natural breast, which finds its subjective attractiveness in the projection and fullness of the upper pole [19]. Breast projection can be enhanced by conization, suturing the flap onto itself (between zones II and III), or de-epithelializing a wedge of skin around the umbilicus and suturing the two pillars together. This possibility depends on the pedicle position and is generally limited in case of a thick abdominal pannus [17].

The fishtail modification serves the objective of increased projection, optimizing volumes within the flap while maintaining a single flap design. By taking advantage of the oblique orientation, the surgeon can comfortably position the flap in line with the anterior axillary line. Then, by splitting the tail of the flap, the upper fin is used to define the lateral IMF and, being fixed to the lateral border of the pectoralis major, to limit the lateral extension of the breast mound as needed. At the same time, excess lateral bulk is shifted toward the central part of the breast and contributes to the core desired projection. In our experience, depending on the degree of orientation and breast width needed, either of the two fins can be used for projection enhancement or lateral IMF definition. The latter is critical for the shape and the projection of the lower pole of the breast [20].

This stepwise approach can be used in case of large and projected contralateral breasts, but can equally suit smaller breasts in thin patients, maximizing the projection and the roundness of the reconstructed mammary cone.

## Conclusion

Proper flap inset is critical to maximize cosmetic outcomes in breast reconstruction. Our systematic approach, focusing on the importance of the lateral IMF and key features of breast aesthetics, can be applied in almost all abdominal-based breast reconstruction procedures and can be easily reproduced to achieve an optimal match with the native breast.

## Supplementary Information

Below is the link to the electronic supplementary material.

Supplementary file1 (MP4 158845 KB)
